# Results of the performance test for quality assessment of personal radiation dosimetry services including the influence of the dosimeter readout frequency

**DOI:** 10.1038/s41598-022-23942-y

**Published:** 2022-11-22

**Authors:** Chen-Ju Feng, Chin-Hui Wu, Yi-Hui Huang, Chien-Hau Chu, Ke-Yu Lien, Yu-Chieh Wang, Shen-Hao Lee, Shih-Ming Hsu

**Affiliations:** 1 Medical Physics and Radiation Measurements Laboratory, National Yang Ming Chiao Tung University, Taipei, Taiwan, Republic of China 112; 2 Department of Biomedical Imaging and Radiological Sciences, National Yang Ming Chiao Tung University, Taipei, 112 Taiwan, Republic of China; 3 Department of Medical Imaging and Radiological Sciences, Tzu-Chi University of Science and Technology, Hualien, Taiwan, Republic of China 970; 4 Department of Radiation Protection, Atomic Energy Commission, New Taipei City, Taiwan, Republic of China 234; 5 Health Physics Division, Institute of Nuclear Energy Research, Taoyuan City, Taiwan, Republic of China 325; 6 Chinese Society of Medical Physics, Taipei, Taiwan, Republic of China 110; 7 Department of Radiation Oncology, Chang Gung Memorial Hospital, Taoyuan City, Taiwan, Republic of China 333

**Keywords:** Biomedical engineering, Techniques and instrumentation

## Abstract

This study was to determine the significance of factors considered for the measurement accuracy of personal dosimeter in dosimetry services such as dosimetry service, irradiation category, years of use and readout frequency. The investigation included management information questionnaire, on-site visit and blind test. The blind test with random selected personal badge was used in inter-comparison of eight dosimetry services, and the test results followed ANSI/HPS N13.11 criteria. This study also analyzed the measurement deviations if they felt in the criteria of ICRP 75 or not. One-way ANOVA tests were used to analyze the significant difference of the measurement deviations in different dosimetry services, irradiation categories, and years of use. Simple linear-regression test was performed for the significance of the prediction model between measurement deviations and readout frequencies. All visited dosimetry services followed the proper statue of basic management and passed the performance check of the tolerance level. The average deviations corresponding to category I, category II deep dose, and category II shallow dose were 6.08%, 9.49%, and 10.41% respectively. There had significant differences of measurement deviation in different dosimetry services (*p* < 0.0001) and irradiation categories (*p* = 0.016) but no significant difference in years of use (*p* = 0.498). There was no significance in the linear-regression model between measurement deviation and badge readout frequencies. Based on the regular calibration of the personal dosimeter, the deviation of the measured value is mainly affected by different dosimetry services and irradiation categories; and there shows no significant influence by years of use and readout frequency.

## Introduction

International Atomic Energy Agency (IAEA) has emphasized that the radiation staff’s occupational dose should consider the as low as reasonably achievable (ALARA) principle^1^. It is essential to monitor the personal radiation dose to ensure a reasonable effective dose limit for radiation workers. In reports of the International Commission on Radiological Protection (ICRP) and International Commission on Radiation Units and Measurements (ICRU), personal dose monitoring should consider deep individual dose, H_p_(10), and shallow individual dose, H_p_(0.07)^1,2^. The integrity of personal dose monitoring requires basic management, practical implementation, and dose record-keeping by accredited dosimetry services^3–6^. The measurement accuracy of personal dosimeters is a critical issue. Thus, the dosimetry services should establish criteria for measurement accuracy^7^.

In the United States, measurement accuracy refers to the American National Standards Institute and the Health Physics Society (ANSI/HPS) N13 series specification for the evaluation of tolerance level on personal dosimeter measurement performance^8,9^. In Europe, the European Radiation Dosimetry Group (EURADOS) conducts routine intercomparison for dosimetry services within multiple countries using the measurement accuracy criteria in each country^10–12^. IAEA also performs comparative studies of dosimetry services in various countries and establishes acceptance limits concerning the deviation between measurement and true irradiation value in ICRP reports^13–16^. Each dosimetry service sends personal dose badges to the National Radiation Standard Laboratory (NRSL) for irradiation following the radiation source category; then then the badges are returned to the original dosimetry services for reading and reporting the personal radiation dose for evaluation. The previous studies related to personal dosimeter measurement accuracy had major discussion on the acceptance comparison between each dosimetry service under current criteria^17,18^. However, it is a lack of discussion on forwarding analysis of the factors like years of use and readout frequency, and whether potentially effecting on the measurement accuracy.

This paper investigated management information of dosimetry services, including qualification recognition, workspace configuration, personnel dose badges, and readout equipment information. The surveyor conducted the blind test on-site^19,20^, visiting each dosimetry service to obtain personal dose badges randomly and sending them to NRSL for irradiation. Later, NRSL sent the badges back to the facilities where they originated to gauge the measurement dose under current criteria^21,22^. Then, the analysis of the influence significance by the significance of factors such as dosimetry service, irradiation category, years of use and readout frequency, on the dose deviation of the personnel dosimeter was performed. The results of this study could determine the significant factors considered for the measurement accuracy constancy of personal dosimeter in dosimetry services, thereby certifying the quality assurance reinforcement of personal dose monitoring of radiation workers.

## Materials and methods

### Questionnaire investigation

This study sent the questionnaire to all eight dosimetry services in Taiwan. The questionnaire was designed with online form (Google LLC, USA). It contained the following questions about personal dose badge information (producers, type of dosimeter, years of use, badge numbers), readout equipment information (equipment models, equipment numbers), and dose record keeping. Table 1 shows the information of the dosimetry services, including badge information and readout equipment information.

**Table 1:** The information within eight dosimetry services in Taiwan.

Badge information					Readout equipment information	
Producer	Model	Type	Place of production	Numbers of dosimetry services	Model	Numbers of using in dosimetry services
Thermo scientific	Harshaw 8814	TLD	USA	4	6600 PLUS 8800 PLUS	3 2
Panasonic	UD-802	TLD	USA/Japan	2	UD-716 UD-7900M	2 1
RADOS	Whole Body	TLD	Germany	1	RE-2000	1
Landauer	UD-874A	OSLD	USA	1	Reader 200	1

*TLD* thermoluminescence dosimeter, *OSLD* optically stimulated luminescence dosimeter.

### On-site visit

The on-site visit was conducted in each dosimetry service. Following the environment condition listed in ANSI/HPS N13.11^23^, the visit examined working space, radiation shielding, environmental control (temperature and humidity), fire alarm system and document storing space. Since the radiation shielding for environment radiation would be consider as an important point of dosimeter storage management, this study used the MiniTRACE CSDF survey meter (Saphymo SAS, Germany) to measure the environmental dose rate in visited facility space.

### Blind test

Each dosimetry service had a random selection of twenty-eight personal dose badges in total, and it was divided into four groups. Each group included six badges for irradiation and one for background calibration. After badges were selected and collected, the badges were sealed with a stamp in one package and sent to NRSL for standard source irradiation. This study employed category I (accidental photon) and category II (general photon) sources for irradiation following ANSI/HPS N13.11. The irradiated personal dose badges were mailed back to the original facilities. The badges must be used to measure the radiation dose readings within three days after the facility receives the package.

The personal dose badge measurement performance was based on ANSI/HPS N13.11 with performance index *Pi*. *Pi* could be obtained by:

1 $$P_{i} = \, \left[ {H_{i} ^{\prime} - H_{i} } \right]/H_{i}$$

where *H*_*i*_*’* is the measurement value reported by the dosimetry services, and *H*_*i*_ is the irradiation value reported by NRSL. With the *Pi* of each badge, the bias, B, could be obtained by:

2 $$B = \frac{{\mathop \sum \nolimits_{i = 1}^{n} P_{i} }}{n}$$

where n is the number of irradiated badges, the Pi and B are used for standard deviation, S, which is obtained by:

3 $$S = \sqrt {\frac{{\mathop \sum \nolimits_{i = 1}^{n} \left( {P_{i} - B} \right)^{2} }}{n - 1}}$$

Finally, the badge measurement performance is defined by:

4 $$B^{{2}} + S^{{2}} \le L^{{2}}$$

where the L is tolerance level. For category I, L is set to be 0.24; for category II, L is set to be 0.3. The category II would consider with deep dose (whole body, H_p_(10)) and shallow dose (skin, H_p_(0.07)). This study also separated the irradiated badges into four intervals by the years of use, including less than 5 years, 5–10 years, 10–15 years, and more than 15 years. Then, the re-calculation of measurement performance was done on the four different time groups.

### Measurement deviation

#### Deviation analysis of irradiated badge

This study also calculated the measurement deviation of the irradiated badge. The measurement deviation could be obtained by:

5 $${\text{Deviation}} = \frac{{H_{i} ^{\prime} - H_{i} }}{{H_{i} }} \times 100\%$$

where *H*_*i*_*’* is the measurement value reported by the dosimetry services, *H*_*i*_ is the irradiation value reported by NRSL. The acceptance level of measurement deviation is based on ICRP report No.75 with the upper and lower limit called ‘trumpet curve’ which defined by Eqs. (6-1) and (6-2)^24,25^.

Upper limit (U.l.) for *H*_*i*_*’/H*_*i*_:

6-1 $${\text{U}}.{\text{l}}.{\text{ for }}\frac{{H_{i} ^{\prime}}}{{H_{i} }} = 1.5\left( {1 + \frac{{H_{O} }}{{2H_{O} + H_{i} }}} \right)$$

Lower limit (L.l.) for *H*_*i*_*’/H*_*i*_:

6-2 $${\text{L}}.{\text{l}}.{\text{ for }}\frac{{H_{i} ^{\prime}}}{{H_{i} }} = \frac{1}{1.5}\left( {1 - \frac{{2H_{O} }}{{H_{O} + H_{i} }}} \right)$$

where *H*_*O*_ is the recording level, it is 0.2 mSv for a general whole-body dosimeter. In this study, the measurement deviations of the irradiated badges corresponding to category I and category II were checked between L.l. and U.l. dose limits.

#### Statistical methods for deviation analysis

One-way ANOVA tests were used to analyze the significant difference of measurement deviation in different dosimetry services, irradiation categories, and years of use. A simple linear-regression test was used to analyze the significance of regression coefficient, R, in the prediction model between measurement deviation (category I, category II deep dose and category II shallow dose) and badge readout frequency^26^. The readout frequencies were separated into five ‘group bins’ for the linear-regression test, including 10, 25, 50, 100, and 150 times in each bin. Data analyses were conducted by SPSS Statistics 24 (IBM, USA). Statistical significance was hypothesized at 0.05.

## Results

### Questionnaire information

**Figure 1:**
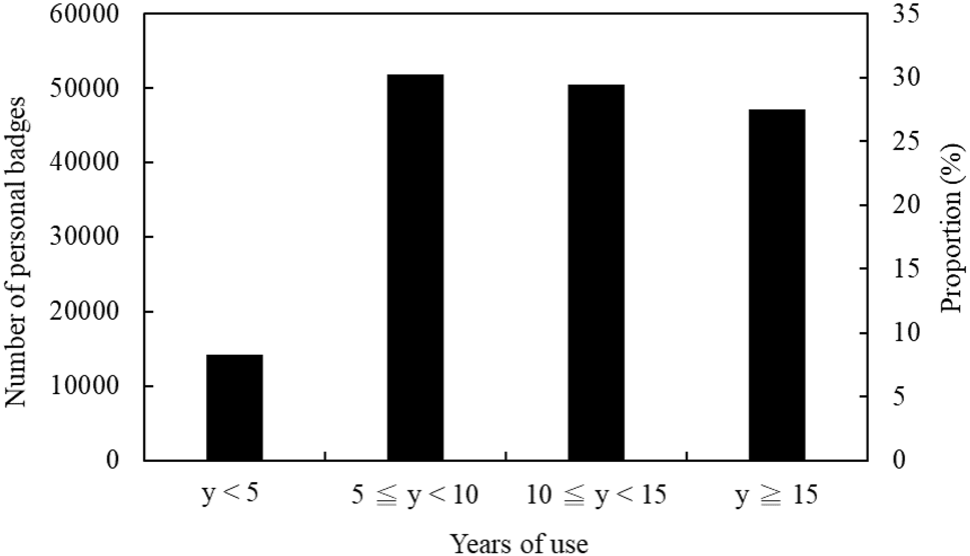
The proportion of personal badges corresponded to four intervals for years of use in Taiwan.

Seven dosimetry services used thermoluminescent dosimeters (TLD), and one service used optical stimulated luminescence dosimeters (OSLD). Each dosimetry service has at least two readout equipment for use. Figure 1 shows the proportion of personal badges within the four intervals for years of use, which are 8.70% (< 5 years), 31.69% (5–10 years), 30.81% (10–15 years), and 28.80% (> 15 years) respectively. All dosimetry services had record keeping for regular monitoring and abnormal dose events.

### On-site visit

All visited dosimetry services in this study followed the environment condition listed in ANSI/HPS N13.11, including proper workspace, radiation shielding, environmental control (temperature and humidity), fire alarm system, and document storing space. The environmental dose rates in all dosimetry service workspaces were less than 0.5 μSv/h on average. All dosimetry services had established record-keeping of backup verification for dose abnormality events.

### Blind test

Table 2 shows the result of the blind test corresponding to categories I and II within each dosimetry service. For category I which irradiated with Cs-137 (0.3 Gy), eight dosimetry services had passed the performance check (L^2^ = 0.058). For category II which irradiated with M150 (30 mSv), eight dosimetry services also had passed the performance check (L^2^ = 0.09). Table 3 shows the results of tolerance level re-calculation corresponding to four intervals for years of use. The number of dosimeters for each year interval (< 5 years, 5–10 years, 10–15 years, and > 15 years) which used for analysis were 34, 47, 22 and 65. The B^2^ + S^2^ values for each interval still appear within the tolerances of the ANSI/HPS N13.11 specification for categories I and II.

**Table 2:** The blind test results corresponded to eight dosimetry services, including accidental (category I) and general photon (category II, deep and shallow).

Participant	B^2^ + S^2^ Value			Pass
	I	II, Deep	II, Shallow	
A	0.007	0.028	0.028	Yes
B	0.002	0.015	0.003	Yes
C	0.004	0.007	0.018	Yes
D	0.007	0.007	0.010	Yes
E	0.005	0.013	0.068	Yes
F	0.004	0.003	0.001	Yes
G	0.013	0.020	0.008	Yes
H	0.008	0.018	0.012	Yes

**Table 3:** The blind test result corresponded to each interval for years of use in this study.

Interval for years of use	B^2^ + S^2^			Pass
	I	II, Deep	II, Shallow	
y < 5	0.007	0.016	0.040	Yes
5 ≦ y < 10	0.007	0.024	0.034	Yes
10 ≦ y < 15	0.008	0.026	0.043	Yes
y ≧ 15	0.004	0.030	0.023	Yes

### Measurement deviation analysis

**Figure 2:**
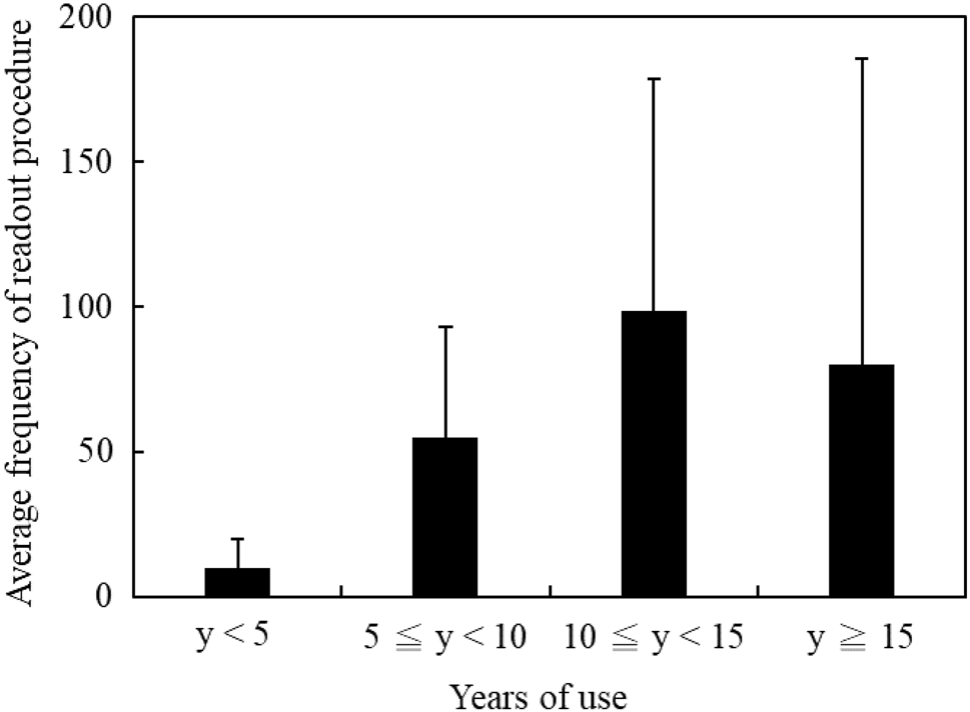
The average frequency of readout procedure in blind test personal badges corresponded to four intervals for years of use.

**Figure 3:**
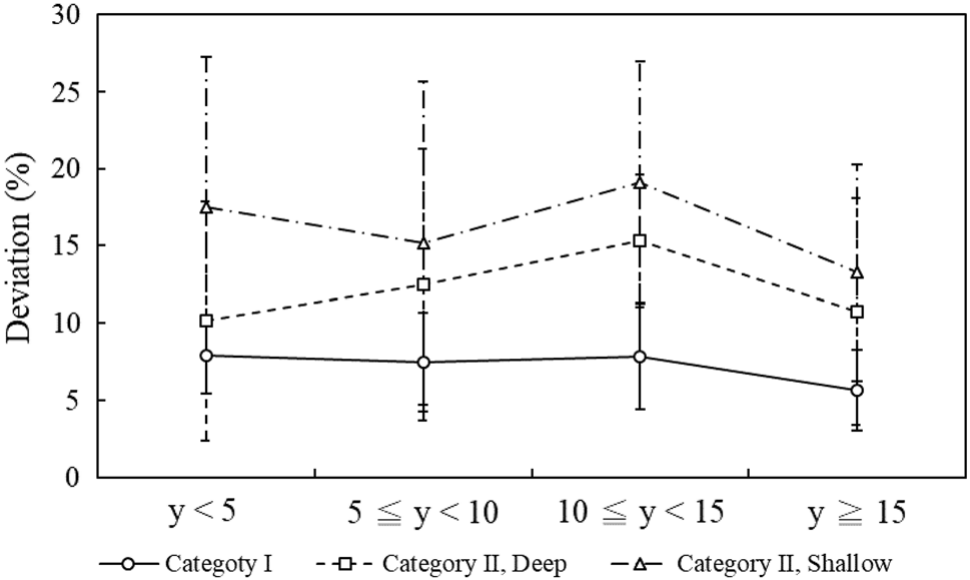
The badge dose deviations within four intervals for years of use which were showed in category I, category II (deep) and category II (shallow).

**Figure 4:**
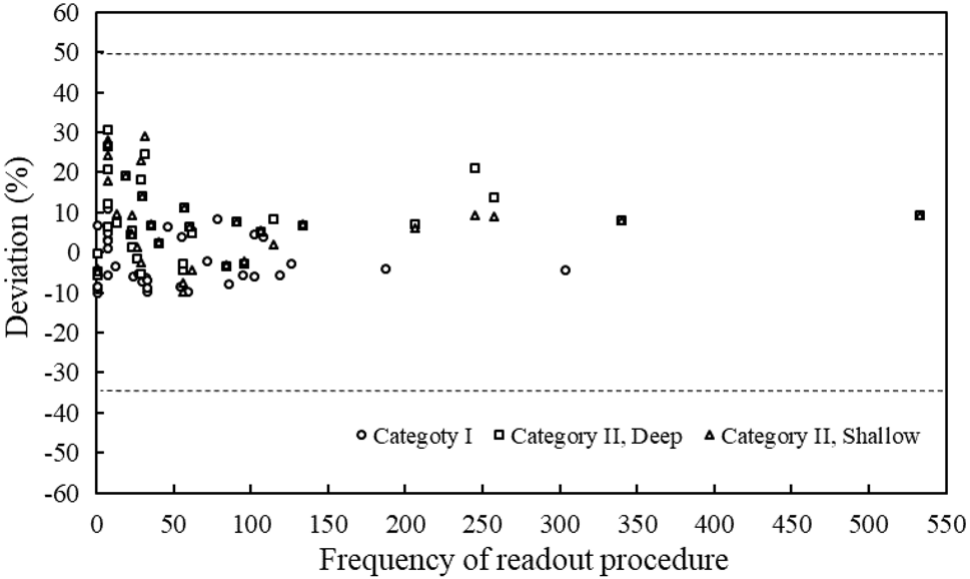
The badge dose deviations corresponded to the frequency of readout procedure, which were showed in categories I, II (deep) and II (shallow).

Figure 2 shows the readout frequencies corresponding to years of use for the selected badges. Approximately half of the selected badges (59.61%) had been used over ten years. The average readout frequency of the chosen badges was 143 times. Figure 3 shows the measurement deviations corresponding to years of use for the irradiated badges. The average deviations corresponding to three categories (category I, category II deep dose, and category II shallow dose) were 6.08%, 9.49%, and 10.41% respectively. Figure 4 shows the measurement deviations corresponding to the readout frequency for the irradiated badges. The overall range of deviations were from − 10.00 to 29.17%. The results of the One-way ANOVA test showed there had significant difference with measurement deviation in: different dosimetry services (*p* < 0.0001) and irradiation categories (*p* = 0.016). There had no significant difference in years of use (*p* = 0.498).

Table 4 shows the result of the linear-regression test. When the readout frequency was used as the prediction model factor of measurement deviation, all readout frequency groupings’ R values had no significance in all group bins for three irradiation categories.

**Table 4:** The results of linear-regression between the frequency of readout procedure and dose deviation within three irradiation categories.

Group bin of readout procedures^†^	I		II, Deep		II, Shallow	
	R	*p*-value	R	*p*-value	R	*p*-value
10	0.040	0.817	0.275	0.105	0.245	0.149
25	0.003	0.985	0.253	0.137	0.233	0.172
50	0.008	0.962	0.269	0.113	0.264	0.120
100	0.016	0.927	0.260	0.126	0.298	0.077
150	0.105	0.542	0.208	0.224	0.193	0.258

^†^The group of readout procedure was based on how many times of readout procedures which was chosen for one bin.

## Discussions

The total number of personal dose badges was about 160 thousand. It was sufficient for personal dose monitoring of radiation workers (about three times as much as the number of workers)^27^. There were more than 90% of in-used personal dose badges used over five years. Also, there were more than 25% of badges over 15 years.

The random selection of badges was proportionally based on the years of usage information offered by each dosimetry service. Thus, it could be sure that the proportion of new and old selected badges was appropriate. The measurement deviations of the irradiated badges corresponding to category I and category II deep dose were between L.l. and U.l. dose limits. According to the result of t-test, the deviation of category II shallow dose was significantly larger than category I (*p* = 0.004). However, the deviations of category II increased comparing to those of category I. It was due to the badge filter correction for the energy dependence of the dosimeter material. This characteristic had more influence on the uncertainty of the radiation energy below 500 keV^28^. Furthermore, the category II shallow dose was calculated from that of category II deep dose by the conversion factor^28,29^. Thus, the deviation uncertainty would be further higher than the category II deep dose.

The sensitivity stability of TLD would be affected by the readout process, thereby it would cause an increase in measurement deviation^30–32^. However, there was no significance in the prediction model when the readout frequency was the factor of linear-regression. This was due to the regular calibration of badges performed by each dosimetry service. Based on the calibration factor, such as effective correction factor/coefficient (ECF or ECC), the dosimetry services routinely excludes the badges that did not meet the calibration criteria. The dosimetry services consisted the stable standard dosimeters/badges as the correction sample, and eliminated the old dosimeters/badges which had high residual signal of glow curve after annealing. Thus, the measurement deviation had no longer highly related to the readout frequency. Furthermore, the R values in category II were higher than those in category I. This showed that the potential correlation with readout frequency for general level dose could be higher than that for accidental level dose. The conversion of measurement value in category II should be more careful. According to the result of linear-regression for category II shallow in this study, it is recommended to establish the measurement stability check for general level dose for the personal badges that have readout frequency over 100 times.

The significant difference in measurement deviations was impacted by the calibration process of each dosimetry service and the irradiation source type of badges. It is necessary for the lead organizer to carry on blind tests regularly or from time to time for taking reference of overall personal dose monitoring quality assurance.

## Conclusion

This study investigated the information of dosimetry services. The results of the bling test showed that the measurement performance of personal badges passed the tolerance level of criteria under random selection mode. Based on the regular calibration of the personal dosimeter, the deviation of the measured value is mainly affected by different dosimetry services and irradiation categories; and there shows no significant influence by years of use and readout frequency. This could determine the significance of factors considered for the measurement accuracy constancy of personal dosimeter in dosimetry services, and reinforce the stability of personal dose monitoring to reach the ALARA principle.

## Data Availability

The datasets generated and/or analyzed during the current study are not publicly available due to [protection of the privacy data for each dosimetry service] but are available from the corresponding author on reasonable request.
